# Serial-multiple mediation of enjoyment and intention on the relationship between creativity and physical activity

**DOI:** 10.3934/Neuroscience.2021008

**Published:** 2021-01-11

**Authors:** Myungjin Jung, Han Soo Kim, Paul D Loprinzi, Minsoo Kang

**Affiliations:** 1 Health and Sport Analytics Laboratory, Department of Health, Exercise Science, and Recreation Management, The University of Mississippi, University, MS 38677, USA; 2 Exercise & Memory Laboratory, Department of Health, Exercise Science, and Recreation Management, The University of Mississippi, University, MS 38677, USA

**Keywords:** creative thinking, emotion, executive function, exercise, prefrontal cortex

## Abstract

The purpose of the present study was to examine a serial-multiple mediation of physical activity (PA) enjoyment and PA intention in the relationship between creativity and PA level (i.e., moderate-to-vigorous PA). A total of 298 undergraduate and graduate students completed a self-reported questionnaire evaluating creativity, PA enjoyment, PA intention, and PA level. Data analysis was conducted using descriptive statistics, Pearson correlation coefficient, ordinary least-squares regression analysis, and bootstrap methodology. Based on the research findings, both PA enjoyment (*β* = 0.06; 95% CI [0.003, 0.12]) and PA intention (*β* = 0.08; 95% CI [0.03, 0.13]) were found to be a mediator of the relationship between creativity and PA level, respectively. Moreover, the serial-multiple mediation of PA enjoyment and PA intention in the relationship between creativity and PA level was statistically significant (*β* = 0.02; 95% CI [0.01, 0.04]). These findings underscore the importance of shaping both cognitive and affective functions for PA promotion and provide additional support for a neurocognitive affect-related model in the PA domain. In order to guide best practices for PA promotion programs aimed at positively influencing cognition and affect, future PA interventions should develop evidence-based strategies that routinely evaluate cognitive as well as affective responses to PA.

## 1. Introduction

Physical inactivity is closely associated with increased risk of numerous health problems, including physiological and psychological diseases (e.g., type 2 diabetes, cancers, obesity, and depression) [1]. Furthermore, lack of physical activity (PA) has resulted in serious medical costs [2]. In contrast, regular participation in PA can help improve physical, social, and cognitive function in various populations [3]–[5]. Of interest to this study is the relationship between PA and cognition, particularly creativity-based cognition. See Figure 1 for our proposed model. In the narrative that follows, we highlight emerging research that supports the delineating pathways of our model. The present study, however, empirically evaluates the robustness of this proposed model.

### 1.1. What is creativity and what brain structures regulate creativity?

Researchers have defined creativity as a broad construct that encompasses the ability to break traditional or obvious patterns of thinking, adopt new or higher order rules, and generate new solutions to specific problems [6]. Creativity requires higher level components of cognitive function, including working memory, inhibitory control, and cognitive flexibility, which is supported by the executive control network [7]. Given that creativity is rooted in executive functions [8], individuals with optimal levels of creativity may have the ability to engage in goal-consistent behaviors while inhibiting goal-inconsistent behaviors. Modern brain research conceptualizes information processing as hierarchically structured, and the most sophisticated cognitive control, such as creativity, is localized at the zenith of that hierarchy, the prefrontal cortex (PFC) [9]. Empirical work in rats supports this by showing that, although rats were trained to acquire a new concept of shifting, prefrontal damage caused the rats to persevere with the previously learned rule, which is indicative of a lack of cognitive flexibility (flexibility is critical in creative thinking [10]). Furthermore, in humans, the ability to shift attentional sets is accompanied by prefrontal activation [11]. As such, this suggests that the PFC may be involved in creativity [12].

The role of PFC in influencing creativity has been investigated by many researchers, and they focused on how modulation of brain activity between executive control and default mode networks within the PFC contributes to the specifics of the production of creative behaviors [13]–[15]. For example, using fMRI to test specific patterns of distributed activation and deactivation in the PFC during improvisation, several studies have suggested that decreased dorsolateral PFC (DLPFC) and increased medial PFC (MPFC) was associated with the creative process [16]–[18]. As discussed elsewhere, the decreased DLPFC may function as an inhibition of conscious volitional control, which in turn, may work directly to facilitate creative behaviors [19]. In contrast, other studies have shown potentially contrasting findings in that an increase in neural activity present in the DLPFC was observed during improvisation [20]. This pattern may be related to top-down regulation [21], which plays an important role in the regulation of prospective memory and creative intelligence [16],[22]. Furthermore, one study that evaluated a computational model of the frontal executive function provided evidence that human frontal lobe function supports integration between reasoning, learning, and creative abilities in open-ended and uncertain situations [23]. Despite this neural plausibility linking various prefrontal function and creativity, there is still no clear evidence of this association, as well as a lack of studies directly examining this association in the PA domain.

### 1.2. Relationship between creativity and mental illnesses

It is widely suggested that there is a relationship between creativity and mental illnesses within the mental health context. In particular, there is a growing interest in the role that creativity is a significant factor in the success of psychiatric treatment [24] and a neurobiological indicator related to mental disorders [25]. A narrative review conducted by Jakovljević [24] highlighted the importance of creative thinking in helping psychiatric patients achieve psychological well-being and a fulfilled life. Further, literature reviews provide suggestive evidence that creative activities can have a therapeutic and protective effect on mental health by promoting a means of self-expression, facilitating problem-solving, increasing motivation, and alleviating stress [24],[26]. More recently, several studies have attempted to identify potential underlying neural mechanisms of creativity and mental disorders [25],[27],[28]. For example, using fMRI, Fink et al. [29] found similar functional brain activity patterns (i.e., decreased deactivation of the right precuneus, a region of the default mode network) during creative cognition in schizotypal subjects and healthy controls. Although widely considered to be linked, creativity and mental disorders are not clearly explained from a neurobiological perspective. As such, additional neuroimaging studies are needed to elucidate this link.

### 1.3. Bidirectional effects of creativity and physical activity (pathway C in Figure 1a)

Accumulating research demonstrates that habitual engagement in PA enhances creativity [30],[31], and this, in turn, may facilitate future PA behavior; thus, the link between PA and creativity may be considered to occur bi-directionally. When it comes to creative individuals' personality traits, scholars generally characterize them as acting according to a plan, self-confident, diligent, determined, and persevering, which is related to functioning in various domains (e.g., school and workplace) [32]–[34]. Based on these unique features of creativity, it is plausible that creative person may be more successful at maintaining a consistent behavior routine in PA settings (see pathway "C” in Figure 1a). This may be due to a number of reasons related to executive function-based creativity, including thorough planning and preparation strategies (e.g., scheduling a certain time each day to participate in PA, having suitable clothing or equipment needed to PA behavior), having strong motivation and concentration to accomplish the PA goal (e.g., setting a feasible goal), and the ability to limit behaviors that interfere with the PA goal (e.g., not skipping PA daily routine despite being tired). Regarding the effects of PA on creativity, PA may lead to improved neurogenesis, synaptic plasticity, and functional connectivity of cortical neuronal networks via augmentation of brain-derived neurotropic factor [35],[36]. Physical activity may also facilitate activation of the hippocampus, which is known to play a critical role in the creative process [37],[38]. Further, regular PA training induces increased neural activity and volume in the prefrontal regions [39]–[41], especially the DLPFC, which is linked to a more complex cognitive process capable of producing novel outcomes [42],[43]. In addition to regular PA, acute PA also increases neural activity in the PFC [44].

### 1.4. Creativity, enjoyment, and physical activity (pathways A and B in Figure 1a)

In addition to creativity potentially being directly influenced by PA behavior [7], creativity may indirectly affect PA behavior via affective responses. Based on a neurocognitive affect-related model in the context of PA, executive function-based cognition (e.g., creativity) may play a key role in shaping and influencing PA-induced affective responses, and this connection, in turn, may promote future PA behavior [45]. For illustrative purposes, given the work highlighting that affective response may be formed from an individual's cognitive interpretations of physiological experiences [46], individuals with enhanced creativity may be more likely to enjoy PA because they have the creative ability to select activities and environments that induce a positive affective response to PA (see pathway “A” in Figure 1a). Further, biological plausibility from neurophysiology and neuroimaging research has shown that regular and moderate-intensity PA elicits the release of the dopamine neurotransmitter, which plays a key role in processing pleasure and reward (motivation), within the MPFC, and this prefrontal dopamine system may evoke emotional as well as a cognitive drive to engage in PA [47],[48]. Importantly, the PFC activated by PA may subserve higher-order cognition, which may, in theory, help change the affective response from previous physiological (arousal) or psychological (valence) experiences of the PA behavior. Considering the above-mentioned physiological mechanisms underlying the function of the PFC, such PA-induced improvements in creativity may foster positive enjoyment of PA, and may ultimately facilitate engagement of PA behavior in the future (see pathway “B” in Figure 1a).

### 1.5. Key drivers of future physical activity: intention (Pathways b_2_ and d_12_ in Figure 1b)

Meanwhile, from the perspective of social psychology theories for comprehending and explaining habitual PA behavior, intentions have been shown to be powerful predictors of PA participation [49] and thus, are regarded as indicators of future PA behavior [50]. Behavioral intention refers to the motivational factors that affect a given behavior where the stronger the intention to perform the behavior, the more likely the behavior will be performed [51]. Similarly, PA intention is defined here as the degree of willingness to continue engaging in PA behavior for at least a few months. Theories such as the Theory of Planned Behavior provide a framework for understanding the determinants of behavioral intentions. This theory posits that an individual's intention to be active is the key determinant of habitual PA behavior, which is determined by their attitude toward the PA behavior, such as harmful-beneficial, good-bad, or unpleasant-pleasant (see pathway “*d_12_*” in Figure 1b) [52]. Consistent with this conceptual framework, several motivational studies in the exercise domain have empirically demonstrated a significant interaction across enjoyment and intentions on exercise persistence, explaining the mediating role of intentions between these two factors [53],[54]. However, affective response of joy and pleasure when exercising does not always translate into maintaining exercise commitment in the long term. Applying to the existing neurocognitive affect-related model of PA behavior, it is reasonable to assume that, as the creative person is commonly energetic, passionate, and persistent (previously mentioned) when acting on a certain behavior, they may feel more interested and motivated to continue participating in PA (see pathway “*a_2_*” in Figure 1b), resulting in higher rates of subsequent PA behavior (see pathway “*b_2_*” in Figure 1b) [55]. Accordingly, further research on this model should take into account PA intention as an independent mediator in effort to disentangle these complex interactions.

Couched within the above, despite the application of this model in the PA domain (e.g., emphasizing on the importance of forming both cognitive and emotional functions for PA promotion), which is expected to contribute to a novel paradigm for the promotion of PA, few empirical studies have evaluated this model. In addition, to our knowledge, studies expanding the neurocognitive affect-related model in the PA context, considering the prediction one outcome to another (i.e., PA enjoyment → PA intention), are scarce. Thus, this study aims to extend the existing model (see Figure 1a) [45] through the serial-multiple mediation of two mediators and empirically test our hypothesized model (see Figure 1b). Ultimately, we hypothesized that creativity would directly associate with PA level (i.e., moderate-to-vigorous physical activity; MVPA) (H1) as well as indirectly influence this outcome via PA enjoyment and PA intention serially (H2–H4).

**Figure 1a.**
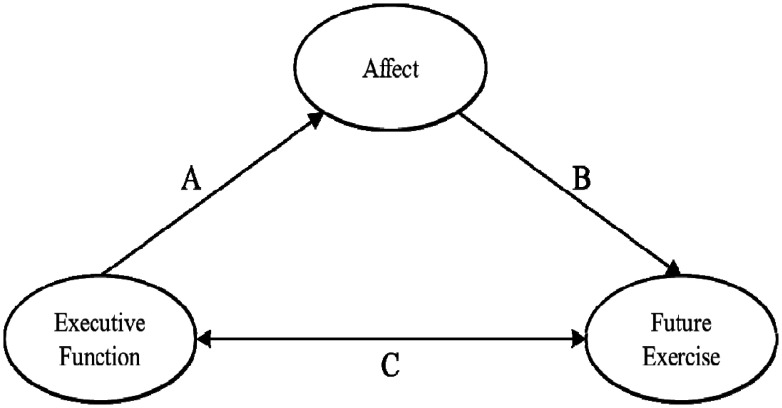
Neurocognitive exercise-based affect-related model [45]

**Figure 1b.**
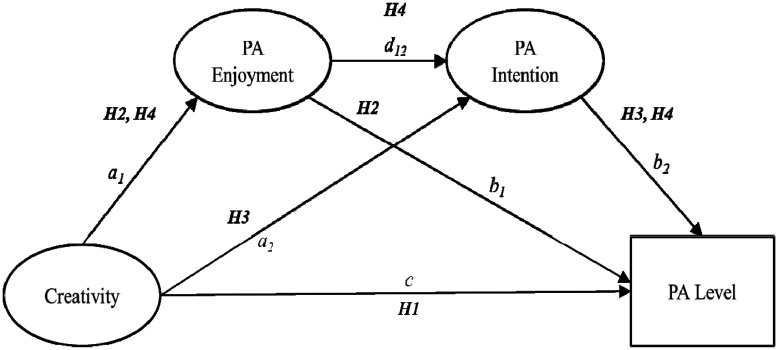
Hypothesized three-path mediation model. (PA: physical activity).

Hypothesis 1. Creativity positively influences on PA level; Hypothesis 2. The relationship between creativity and PA level is mediated by PA enjoyment; Hypothesis 3. The relationship between creativity and PA level is mediated by PA intention; Hypothesis 4. The relationship between creativity and PA level is serially mediated by PA enjoyment and PA intention.

## 2. Materials and methods

### 2.1. Participants and procedures

To accomplish the purpose of this study, an online survey created using Qualtrics was administered to a University-affiliated young adult population after approval of the detailed research protocol and questionnaire by the Institutional Review Board. The sample for this study was collected in the following steps. The current study utilized a convenience sampling method. The email addresses of undergraduate and graduate students were obtained from the authors' University. After retrieving the email addresses, a URL link for the survey was sent to 2,040 prospective respondents over a 4-week period and 310 questionnaires were returned (response rate: 15.2%) prior to COVID-19 pandemic. A total of 298 participants completed the online survey, except for 12 invalid data with unclear or absent responses. The sample size of this study was met the minimum criteria for conducting multivariate analysis, as the total number of participants was more than 200 [56].

### 2.2. Measures

Participants completed a 30-item survey assessing creativity, PA enjoyment, PA intention, and PA level.

Creativity. The self-evaluated creativity instrument utilized a short form of the Kaufman Domains of Creativity Scale (K-DOCS) [57]. It included 20 items measuring five domains of creativity relevant to college student populations. The five domains were Self/Everyday, Scholarly, Performance, Mechanical/Scientific, and Artistic. An example item evaluating Self/Everyday creativity is “Teaching someone how to do something.” An example item evaluating Scholarly creativity is “Arguing a side in a debate that I do not personally agree with.” An example item measuring Performance creativity is “Playing music in public.” An example item assessing Mechanical/Scientific creativity is “Solving math puzzles.” and lastly, an example item for Artistic creativity is “Appreciating a beautiful painting.” The measurement was based on a five-point Likert-type scale, which ranged from 1 (much less creative) to 5 (much more creative). Item ratings were summed for each distinct domain, with higher scores indicative of higher domain creativity. The short version of the K-DOCS has been reported to have acceptable internal consistency. In an independent sample, Cronbach alpha coefficients were 0.69 for self/everyday, 0.67 for scholarly, 0.82 for performance, 0.77 for mechanical/scientific, and 0.75 for artistic creativity [58]. Internal consistency of the present sample, across these 5 respective domains, is similar to previous research, and was 0.65, 0.68, 0.85, 0.85, and 0.81; notably, the three assumptions of Cronbach's alpha (unidimensionality, uncorrelated error scores, and items' true scores differ by an additive constant [59]) were confirmed for this, and all, internal consistency analyses.

Physical Activity Enjoyment. In the PA enjoyment section, we used the modified Physical Activity Enjoyment Scale (PACES) to measure enjoyment of the PA. To reflect the study aims, 5 items were chosen from the original 18-item [60]. The items are preceded by the phrase “When I am physically active...”. Example items include “I hate it,” “I like it,” “It's a lot of fun,” “I feel bad physically while doing it,” and “I am not at all frustrated by it.” Each item was based on a 7-point rating scale from 1 (strongly disagree) to 7 (strongly agree), and two items are reverse scored. Total responses were summed to give a score ranging from 5 to 35, with higher PACES scores indicating greater levels of enjoyment. According to previous research [61], these 5 items have shown strong internal consistency in a young adult sample (Cronbach's alpha ≥0.78). In the present sample, internal consistency, measured by Cronbach's alpha, was 0.87.

Physical Activity Intention. Intention to continue engaging in PA was evaluated using three items, which was developed by McAuley and Courneya [62]. Following the stem, ‘these questions focus on your PA plans for the next 4 months', a sample item is “I intend to participate in physical activity regularly during the next 4 months.” Each item used a 7-point Likert scale from 1 (strongly disagree) to 7 (strongly agree), and scores were calculated by averaging the 3 items of the scale. This scale has been demonstrated to have acceptable validity (factorial validity = 0.93) and reliability (Cronbach's alpha = 0.89) in an independent sample [63]. For the present sample, the internal consistency across the 3 items was 0.86.

Physical Activity Level. PA level was assessed from a two-item Physical Activity Vitals Sign (PAVS) survey. The first question asked participants to indicate the number of days in a typical week they engaged in MVPA. The second question asked participants to indicate the average time spent in MVPA on these days. The products of these two items were calculated to represent the weekly engagement in MVPA. This questionnaire has demonstrated evidence of validity in an independent sample (concurrent validity = 0.71) [64].

Additional Assessments. Demographic information (e.g., age, gender, race/ethnicity, education level, and grade point average) were gathered from self-reported items. Body mass index (BMI) was estimated from self-reported weight in kilometers divided by self-reported height in meters squared. Additionally, these background variables were used as covariates to minimize potential confounding.

### 2.3. Data analysis

SPSS 26.0 and AMOS 26.0 were utilized for the analyses. First, descriptive statistics were used to describe and summarize the collected data. Pearson correlations among factors were subsequently conducted and confirmed. To evaluate the reliability and validity of the latent constructs (i.e., creativity, PA enjoyment, and PA intention), Cronbach's alpha, composite reliability (CR), and average variance extracted (AVE) were calculated. Specifically, Cronbach's alpha is a measure used to evaluate the reliability, or internal consistency, of a set of scale items. Composite reliability is a measure of reliability and internal consistency of the measured variables representing a latent construct, which is likely to be a less biased estimate of reliability than Cronbach's alpha. To assess the convergent validity of constructs, AVE was computed. Average variance extracted is the average percentage of variation explained by items in a construct [65]. The discriminant validity was evaluated by comparing the square root of AVEs and construct correlations [66].

We conducted multiple confirmatory factor analysis (CFAs) using maximum likelihood method (MLM) to verify the second-order construct (i.e., creativity) as well as the full measurement model. Confirmatory factor analysis is used to test a priori hypotheses about associations between observed variables and latent constructs, and to evaluate the validation of construct and the measurement model in path or structural analysis [67]. For example, in case of the second-order construct, CFA is employed to assess whether the theorized main construct in a study loads into certain number of underlying sub-constructs. When conducting CFA, the most commonly used method of parameter estimation is the MLM based on the assumption of multivariate normality. This method is utilized to maximize probability distributions and parameters that best describe the observed data [68].

In order to assess the goodness-of-fit measurement model, we utilized various goodness-of-fit indices, including the chi-square (χ^2^), the Comparative Fit Index (CFI), the Tucker-Lewis Index (TLI), the Root Mean Square Error of Approximation (RMSEA), and the Standardized Root Mean Square Residual (SRMR). In general, CFI and TLI values that are equal to or greater than 0.90 indicate a good fit to the data [56]. These fit indices evaluate the extent to which the observed data fit the tested (e.g., measurement or hypothesized) model. Particularly, CFI indicates the fit of the target model to the fit of the tested model by comparing the observed model to a null model [69], whereas TLI indicates the fit of the target model to the fit of the tested model while considering the degrees of freedom of the specified model and the degrees of freedom of the independence model [70]. The RMSEA and SRMR evaluate discrepancies between observed and predicted covariances, with a cutoff value of .06 for RMSEA and .08 for SRMR [56].

Statistical significance of our hypothesized model was analyzed through the PROCESS Macro for SPSS developed by Hayes [71], using ordinary least-squares regression and bootstrap methodology. Bootstrapping is a non-parametric test that involves thousands of sampling observations that are randomly replaced from the data set to calculate the desired statistic in each resample [72]. The basic concept of Hayes' approach is that the statistical significance of the indirect mediating effects of variables upon the bootstrap method is assessed based upon whether the point estimate of the mediator is zero within a 95% bootstrap confidence interval. Accordingly, a variable with a non-point estimate within the zero-interval is regarded statistically significant. In this study, 95 percent confidence intervals were employed and 10,000 bootstrapping re-samples were performed using the serial-multiple mediation analysis (i.e., model 6). The strength of this approach is that it enables isolation of each mediator's indirect effect and further, it allows investigating the indirect effect passing through both of the mediators in a series.

## 3. Results

### 3.1. Demographic characteristics

Table 1 displays the characteristics of the sample. Participants, on average (SD), were 23.2 (4.9) years and were predominately female (65.1%), non-Hispanic white (65%), and undergraduate (69.3%). The average time spent in MVPA, BMI, and GPA were 269.3 min/week, 24.5 kg/m^2^, and 3.6 (out of 4.3), respectively.

### 3.2. Measurement model

Confirmatory factor analysis for second-order measurement was conducted prior to that for the full measurement model. First, 3 items for creativity were excluded due to low factor loadings (i.e., <0.50) [56], and the remaining 17 items were analyzed for CFA (see Table 2). The result showed an acceptable model fit (χ^2^ = 125.41, *df* = 67, *p* < 0.001, CFI = 0.97, TLI = 0.96, SRMR = 0.05, RMSEA = 0.05). Second, CR and AVE values of each latent construct exceeded the suggested levels of 0.70 and 0.50 [56], respectively. Third, correlations among sub-constructs were calculated to evaluate multi-collinearity or singularity issues, but none of the relationships exceeded the cut-off point of 0.85 [73]. Fourth, all the square roots of the AVEs exceeded corresponding construct correlations, indicating acceptable discriminant validity.

In terms of the full measurement model, including the latent constructs (i.e., creativity, PA enjoyment, and PA intention), the result showed a good model fit (χ^2^ = 429.11, *df* = 296, *p* < 0.001, CFI = 0.96, TLI = 0.96, SRMR = 0.05, RMSEA = 0.04). Both CR and AVE values exceeded the suggested levels of 0.70 and 0.50, respectively, indicating acceptable reliability and validity [56]. The correlations did not exceed the cut-off point of 0.85 (see Table 3), indicating that multi-collinearity or singularity issues were not found. To test discriminant validity in the final measurement model, we compared squared inter-construct correlation (SIC) values with AVE values for each latent constructs. Consequently, all AVE values were higher than the SIC values for each construct, supporting the discriminant validity in the final measurement model [66].

### 3.3. Hypothesis testing

**Figure 2.**
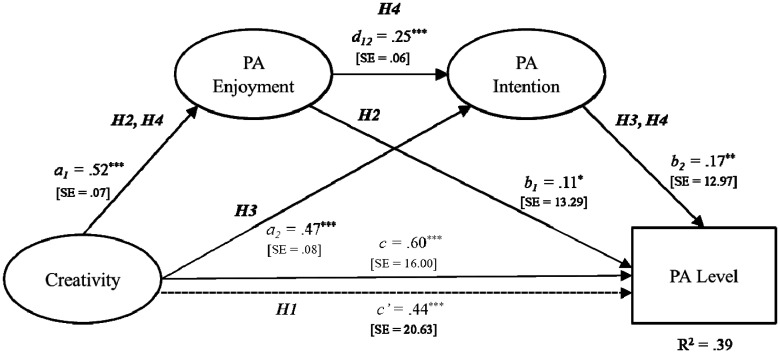
Hypotheses testing through a serial-multiple mediation model. (Serial-multiple mediation of PA enjoyment and PA intention in the relationship between creativity and PA level with standardized beta coefficient. PA: physical activity. *p < 0.05. **p < 0.01. ^***^p < 0.001).

To determine the serial-multiple mediation of PA enjoyment and PA intention in the relationship between creativity and PA level, this study calculated all standardized path coefficients, simultaneously controlling for above-stated variables, through the mediation analysis. Obtained direct path coefficients are displayed in Figure 2, and the comparison of the indirect effects of creativity on PA level through PA enjoyment and PA intention is included in Table 4.

**Table 1.** Participant characteristics.

Variable	Point Estimate	*SD*
Age, mean years	23.2	4.9
% Female	65.1	
Race-Ethnicity, %		
Mexican American	4.6	
Other Hispanic	4.9	
Non-Hispanic white	65.0	
Non-Hispanic black	11.4	
Other	14.1	
Education level, %		
Undergraduate	69.3	
Graduate	30.7	
BMI, mean kg/m^2^	24.5	5.3
GPA, mean	3.6	0.4

Note: All point estimates are arithmetic means. BMI: body max index; GPA: grade point average.

**Table 2.** The finalized survey questionnaire.

Variable	Items
Creativity	
Self/Everyday	Maintaining a good balance between my work and my personal life
	Understanding how to make myself happy
Scholarly	Responding to an issue in a context-appropriate way
	Arguing a side in a debate that I do not personally agree with
	Being able to offer constructive feedback based on my own reading of a paper
Performance	Making up lyrics to a funny song
	Composing an original song
	Spontaneously creating lyrics to a rap song
	Playing music in public
Mechanical/Scientific	Writing a computer program
	Solving math puzzles
	Taking part machines and figuring out how they work
	Building something mechanical (like a robot)
Artistic	Sketching a person or object
	Doodling/drawing random or geometric designs
	Making a scrapbook page out of my photographs
	Appreciating a beautiful painting
PA enjoyment	When I am physically active, I hate it (R)
	When I am physically active, I like it
	When I am physically active, it's a lot of fun
	When I am physically active, I feel bad physically while doing it (R)
	When I am physically active, I am not at all frustrated by it
PA intention	I intend to participate in physical activity regularly during the next 4 months
	I intend to participate in physical activity as much as I can every week during the next 4 months
	I intend to participate in physical activity at least three times per week over the next 4 months
PA level	On average, how many days per week do you engage in moderate to strenuous exercise?
	On average, how many minutes per day do you engage in exercise at that level?

Note: R: reverse-scored item.

**Table 3.** Descriptive statistics, CR, AVE, and correlations.

Variable	*M*	*SD*	CR	AVE	1 (a)	2 (b)	3 (c)	4 (d)	(e)
1. Creativity	3.6	0.6	0.72	0.52	1				
a. Self/Everyday	3.8	0.8	0.81	0.70	(1)				
b. Scholarly	3.9	0.7	0.78	0.54	(0.20**)	(1)			
c. Performance	3.5	1.0	0.87	0.69	(0.45**)	(0.14**)	(1)		
d. Mechanical/Scientific	3.2	1.0	0.87	0.69	(0.39**)	(0.17**)	(0.57**)	(1)	
e. Artistic	3.6	0.9	0.82	0.60	(0.33**)	(0.22**)	(0.38**)	(0.47**)	(1)
2. PA enjoyment	6.0	1.0	0.88	0.59	0.52**	1			
3. PA intention	5.5	1.5	0.86	0.68	0.60*	0.50**	1		
4. PA level (min/week)	269.3	131.2	-	-	0.60**	0.43**	0.49**	1	-

Note: CR: composite reliability; AVE: average variance extracted; PA: physical activity. *p < 0.05. **p < 0.01.

**Table 4.** Indirect effects of creativity on PA level through PA enjoyment and PA intention.

Indirect effects	Product of coefficients		95% CI^a^		Figure path
	Coefficient	SE	Lower	Upper	
Total indirect associations	0.16	0.04	0.08	0.24	
Creativity → PA.E → PA level	0.06	0.03	0.003	0.12	*a_1_b_1_*
Creativity → PA.I → PA level	0.08	0.03	0.03	0.13	*a_2_b_2_*
Creativity → PA.E → PA.I → PA level	0.02	0.01	0.01	0.04	*a_1_b_1_ + d_12_b_2_ + a_2_b_2_*

Note: PA.E: physical activity enjoyment; PA.I: physical activity intention; PA: physical activity. ^a^10,000 bootstrap samples for 95% bootstrap confidence intervals.

**Table 5.** Direct effects among variables.

Variable	PA enjoyment (First mediator)				PA intention (Second mediator)				PA level (Outcome variable)			
	Product of coefficients		95% CI		Product of coefficients		95% CI		Product of coefficients		95% CI	
	Coefficient	SE	Lower	Upper	Coefficient	SE	Lower	Upper	Coefficient	SE	Lower	Upper
Creativity	0.53 (.75)	0.07	0.61	0.89	0.46 (0.72)	0.08	0.56	0.89	0.45 (154.00)	20.83	113.00	195.00
PA enjoyment	-	-	-	-	0.25 (.27)	0.06	0.16	0.38	0.11 (27.17)	13.34	0.92	53.41
PA intention	-	-	-	-	-	-	-	-	0.16 (34.66)	13.09	8.88	60.43
Other covariates
Age	0.02 (.002)	0.01	−0.01	0.01	0.03 (0.003)	0.01	−0.01	0.01	0.05 (1.36)	1.26	−1.11	3.83
Gender (female = Ref)	−0.04 (−0.04)	0.06	−0.16	0.08	−0.01 (−0.01)	0.06	−0.12	0.10	−0.04 (−11.28)	12.87	−36.60	14.05
Race-Ethnicity (White = Ref)
Mexican American	−0.02 (−0.04)	0.13	−0.30	0.22	−0.10 (−0.28)	0.13	−0.54	−0.02	−0.05 (−28.02)	29.15	−85.40	29.36
Other Hispanic	−0.01 (−0.01)	0.13	−0.27	0.25	−0.02 (−0.06)	0.13	−0.31	0.19	−0.02 (−13.65)	28.49	−69.73	42.43
Non-Hispanic black	0.05 (0.09)	0.09	−0.09	0.26	0.04 (0.08)	0.09	−0.10	0.25	−0.03 (−12.01)	19.79	−50.96	26.95
Other	0.03 (0.05)	0.09	−0.12	0.21	0.001 (0.001)	0.08	−0.17	0.17	0.08 (31.61)	18.68	−5.15	68.37
Education level (graduate = Ref)	−0.02 (−0.02)	0.06	−0.14	0.11	−0.01 (−0.02)	0.06	−0.14	0.11	−0.01 (−3.07)	13.87	−30.36	24.23
BMI	0.04 (0.004)	0.01	−0.01	0.02	−0.04 (−0.004)	0.01	−0.02	0.01	−0.05 (−1.20)	1.17	−3.51	1.10
GPA	−0.02 (−0.03)	0.07	−0.18	0.11	0.01 (0.02)	0.07	−0.12	0.16	−0.07 (−22.69)	15.81	−53.80	8.43

Note. Direct effects among variables with standardized beta coefficient (unstandardized beta coefficient). PA: physical activity; Ref: reference; BMI: body max index; GPA: grade average point.

As shown in Figure 2, analysis of H1 tested the total effect of creativity on PA level. The result of the regression analysis indicated the hypothesized effect to be statistically significant (*β* = 0.60, SE = 16.00, *p* < 0.001), supporting H1. For H2, the results showed that creativity influenced PA level indirectly through the mediation of PA enjoyment (*β* = 0.06, SE = 0.03; 95% CI [0.003, 0.12]), supporting H2. H3 states that the relationship between creativity and PA level is mediated by PA intention. The significance test entailed estimation of an indirect of creativity through PA intention on PA level. According to the results, creativity affected PA level indirectly through the mediation of PA intention (*β* = 0.08, SE = 0.03; 95% CI [0.03, 0.13]). Thus, H3 is supported. Lastly, H4 states that the relationship between creativity and PA level is serially mediated by PA enjoyment and PA intention. The indirect effect of creativity on PA level through the serial-multiple mediation of PA enjoyment and PA intention was examined and the result was found to be statistically significant (*β* = 0.02, SE = 0.01; 95% CI [0.01, 0.04]), supporting H4. The results also showed that none of the covariates were associated with the study variables, all *p*'s > 0.05 (see Table 5).

## 4. Discussion

In the theoretical framework of the neurocognitive affect-related model of PA [45], this model hypotheses that executive function-related cognition is not only directly associated with habitual engagement in PA but also indirectly associated with future PA via affective response to PA. In the present study, we provide theoretical support for this model. Specifically, we first found that creativity positively influenced on PA level. As expectedly, individuals with higher levels of creativity may have a greater ability to maintain appropriate behavioral patterns and mental state to achieve their future goals, whereas individuals with lower levels of creativity may have difficulty maintaining a consistent daily routine [74]. In another direction, emerging work suggests that PA (or exercise) may help to improve creativity [75]–[77]. Although the mechanism(s) to illustrate this potential relationship is still unclear, PA may influence creativity through brain mechanisms related to neural structure and function [47],[78]–[81]. Relatedly, PA is suggested to alter neuroanatomical correlates of higher-order cognitive functions relevant to creativity, such as memory and executive function [80], and advance neural growth and development in critical brain areas linked with creative mentation [81]. In terms of neurochemical nature, perhaps PA is also capable of inducing synaptic release of catecholamine in the PFC, and in turn, this PA-related modulation of catecholamine release may partially mediate cognitive creativity performance [47],[79]. Per the inverted U-shaped hypothesis [82], moderate-intensity PA may enhance PFC-dependent cognition (i.e., tasks that rely on executive functioning, including creativity), which perhaps is due to increases in prefrontal D1-dopaminergic and a2A-adrenoreceptors by dopamine and norepinephrine, respectively [83]. In addition to creativity, affective judgement plays an important role in engaging in current behavior and predicting future behavior. Affective judgement is considered to be based on one's affective response to a certain behavior, drawing conclusions as to whether this behavior is either of value or not of value. As such, if the behavior is regarded as valuable, people are likely to feel a positive affective response to the behavior, and ultimately this may facilitate future behavioral engagement [45],[84]. These findings are in alignment with previous experimental work [85], which demonstrated that greater performance on a cognitive task was associated with favorable affective response to exercise, and in turn, this positive affect was associated with promoting current and future PA behavior. Taken together, there may be utility in enhancing creativity to increase the perceived enjoyment of PA and the amount of PA.

Notably, such complex interrelationships can be potentially explained by functional neuroanatomy and chemical neurotransmission aspects. As discussed thoroughly elsewhere [86], the PFC is not a single unit, that is, it is functionally classified into ventromedial (VMPFC) and dorsolateral aspects (DLPFC). Functional imaging studies have shown that executive function-based cognition is heavily dependent on the DLPFC, whereas emotional function is implemented in the VMPFC [87]. Given the DLPFC is closely interconnected with the VMPFC, PFC activation may help integrate highly cognitive and affective information processing. Applied within the context of PA, PA-induced prefrontal activity may improve executive function-based cognition, and in turn, this effect may help integrate feelings of pleasure and arousal by favorably interpreting previous PA experiences as well as the expected health effects of PA behavior in the future. Furthermore, the mesocorticolimbic dopaminergic system, originating from the ventral tegmental area, has projections to the PFC [88], which may play an important role in executive functioning-related processes as well as neural responses to emotion (e.g., motivation, stress, and reward) [89],[90]. Indeed, increased dopamine concentrations in the brainstem have been shown to facilitate cognitive and affective improvements [83],[91].

An additional finding in our study is that we refined the existing neurocognitive affect-related model by showing that intention to continue engaging in PA may play a key mediating variable in the relationship between creativity and PA level; in addition, PA enjoyment and PA intention mediated this relationship serially. These findings are relevant because previous studies have indicated that enjoyment and behavioral intention are considered together as major drivers of PA participation [49]. Given the theoretical sequence whereby emotional outcome (i.e., enjoyment) could predict a cognitive outcome (i.e., intention to perform the behavior in the future), within the PA context, higher levels of enjoyment of PA may predict higher levels of intention to perform PA behavior, which in turn may lead to higher levels of future PA engagement [50]. This can be considered as an additional implication supplementing the interrelationships between creativity, PA affect, and PA behavior that future PA behavior can be influenced by various factors, including enjoyment and intention. Although this is a welcome addition to the literature given the lack of research into the proposed model in the PA context, no studies as of yet have empirically tested how the two mediators function together in this relationship. Besides, considering not only creativity but also other cognitive activities are associated with executive function [92], we should not imply that PA directly promotes creativity itself. It is plausible that capacity of learning or knowledge, which is closely related to creativity, may also likely to be modulated by physical functions [93],[94]. Thus, this is an area in need of a follow-up, longitudinal study.

Despite the fact that we tested this theory-based model through the serial-multiple mediation model, there are several limitations in the present study. The first limitation is related to the self-reported assessment of the evaluated constructs. Although the appropriate survey items were selected and adapted for the current study, several items were removed due to poor psychometric properties. Relatedly, any proposed cause-and-effect associations among variables are unable to be determined because of the cross-sectional nature of the present study. Further, we did not utilize an objective measure of PA. Given that subjectively self-report PA measurements are prone to substantial measurement errors, such as over-estimating of PA levels, compared to PA objectively measured with devices (e.g., accelerometer), future work is needed that objectively measures PA in order to more accurately evaluate PA behavior. Similarly, as BMI was estimated by self-reported weight and height, we should carefully interpret the observed covariate effects. Another limitation includes our homogenous sample, limiting the study's generalizability. Although the purpose of the study was to empirically test our hypothesized model, additional research should continue to evaluate this model using other populations, including young children. For example, it would be interesting to conduct research on this topic in a sample of young children, considering that children have plenty of opportunities to foster the ability to be creative as well as the notion that critical thinking and creative problem-solving skills are primary goals for children's development [95]. In spite of these limitations, strengths of this study include its novelty. No research to date has used this analytical approach to evaluate these complex interrelationships.

## 5. Conclusions

In conclusion, consistent with our proposed model, we demonstrated that creativity was associated with higher PA enjoyment and PA intention, which relate to higher levels of PA level. If our findings are supported with future research, then these findings may contribute to developing and refining this model, which may help cultivate strategies to promote PA behavior among a wide variety of populations.
